# miRCOVID-19: Potential Targets of Human miRNAs in SARS-CoV-2 for RNA-Based Drug Discovery

**DOI:** 10.3390/ncrna7010018

**Published:** 2021-03-02

**Authors:** Tanvir Alam, Leonard Lipovich

**Affiliations:** 1 College of Science and Engineering, Hamad Bin Khalifa University, Doha 34110, Qatar; 2 College of Medicine, Mohammed Bin Rashid University of Medicine and Health Sciences, 505055 Dubai, United Arab Emirates

**Keywords:** COVID-19, SARS-CoV-2, miR-122, HCV, coronavirus, microRNA, miRNA, anti-miR, antagomir, RNA therapeutics, Miravirsen

## Abstract

Sense-antisense interactions of long and short RNAs in human cells are integral to post-transcriptional gene regulation, in particular that of mRNAs by microRNAs. Many viruses, including severe acute respiratory syndrome coronavirus 2 SARS-CoV-2 (the causative agent of coronavirus disease 2019, COVID-19), have RNA genomes, and interactions between host and viral RNAs, while known to be functional in other viral diseases, have not yet been investigated in COVID-19. To remedy this gap in knowledge, we present miRCOVID-19, a computational meta-analysis framework identifying the predicted binding sites of human microRNAs along the SARS-CoV-2 RNA genome. To highlight the potential relevance of SARS-CoV-2-genome-complementary miRNAs to COVID-19 pathogenesis, we assessed their expression in COVID-19-relevant tissues using public transcriptome data. miRCOVID-19 identified 14 high-confidence mature miRNAs that are highly likely to interact with the SARS-CoV-2 genome and are expressed in diverse respiratory epithelial and immune cell types that are relevant to COVID-19 pathogenesis. As a proof of principle, we have shown that human miR-122, a previously known co-factor of another RNA virus, the hepatitis C virus (HCV) whose genome it binds as a prerequisite for pathogenesis, was predicted to also bind the SARS-CoV-2 RNA genome with high affinity, suggesting the perspective of repurposing anti-HCV RNA-based drugs, such as Miravirsen, to treat COVID-19. Our study is the first to identify all high-confidence binding sites of human miRNAs in the SARS-CoV-2 genome using multiple tools. Our work directly facilitates experimental validation of the reported targets, which would accelerate RNA-based drug discovery for COVID-19 and has the potential to provide new avenues for treating symptomatic COVID-19, and block SARS-CoV-2 replication, in humans.

## 1. Introduction

Severe acute respiratory syndrome coronavirus 2 (SARS-CoV-2), belonging to the genus Betacoronavirus, was first identified in China, where it was initially associated with widespread severe human illness in late 2019, and has subsequently rapidly emerged into a global pandemic of the novel, highly transmissible, frequently severe respiratory illness (coronavirus disease 2019, i.e., COVID-19) that it causes. The unprecedented health and socioeconomic effects of this global disaster, which are currently mitigated solely by non-pharmaceutical interventions [1], urgently warrant the development of new infection control and treatment measures [2,3]. SARS-CoV-2 is the seventh coronavirus known to infect humans. Two other recently-emerged betacoronaviruses (SARS-CoV and Middle East respiratory syndrome coronavirus (MERS-CoV)) can cause severe human disease, but have only been observed in a one-time event and in limited outbreaks respectively, while four commonly-circulating coronaviruses (the betacoronaviruses OC43 and HKU1, as well as the alphacoronaviruses NL63 and 229E) are globally endemic and mainly cause mild seasonal common-cold illnesses. Coronaviruses are positive-strand RNA viruses. While coronaviruses have become the subject of enormous attention and grave concern as a consequence of the current worldwide COVID-19 public-health crisis, other viruses of extraordinary public-health significance also have RNA genomes, including the dengue viruses, filoviruses (including Ebola), and hepatitis C virus (HCV) [2].

MicroRNAs (“miRNAs”) are processed, endogenous, small RNA molecules that serve as fundamental post-transcriptional repressors in nearly all normal development and disease processes in metazoan cells. The major endogenous mechanism of microRNA action entails the binding of microRNAs to their protein-coding messenger-RNA (mRNA) cellular targets. The RNA–RNA binding of a microRNA and its target is sequence-specific, and—although it does not require perfect sequence complementarity—is based on sense-antisense, Watson-Crick base pairing between the microRNA seed sequence and a cognate complementary sequence in the target mRNA, typically in the target’s 3′ untranslated region. This binding leads to the inactivation of the corresponding mRNAs through the well-characterized RNA-induced silencing complex (RISC) pathway [4], reducing or eliminating protein translation from those mRNA templates. Although miRNAs normally bind mRNAs, they can also form RNA-RNA interactions with other RNAs in the cell, including non-coding RNAs and—if the cell is infected with a virus—the viral genome (if it is also an RNA), as well as pathogen-encoded mRNA and other RNA transcripts. The potential for these host-pathogen RNA–RNA interactions to modulate both the progression of and the responses to infection is now increasingly recognized [5,6].

Antago-miRs (synonym: “anti-miRs”) are modified chemical backbone oligonucleotide drugs that are designed based on the principle of sequence complementarity with the aim to pair with, and thus inactivate, specific cellular microRNAs, thereby blocking these microRNAs from carrying out their (typically pathogenesis-associated) cellular roles (such as the inappropriate suppression of mRNAs whose depletion leads to the development of the disease being treated). Drugs of this class have recently been deployed against miR targets in humans to treat diseases that are caused by the deregulation of endogenous miRNA–mRNA interactions [7,8]. More recently, anti-miRs have also been considered as a novel tool to attack disease-associated interactions between human cellular miRNA and viral (rather than host) RNA genomes, as well as virally encoded transcripts during virus infections. Human hepatocytes endogenously express the microRNA miR-122, which base pairs with the HCV genomic RNA and serves as an essential cofactor for HCV replication. Understanding the essential role of miR-122 as an HCV cofactor led to the development of Miravirsen, which is the first RNA-oligonucleotide drug to specifically target a virus–host RNA–RNA interaction [9]. Miravirsen is an anti-miR complementary to miR-122. By binding to miR-122, Miravirsen prevents miR-122 from hybridizing to the HCV RNA genome, hence depriving HCV of its essential cellular cofactor and blocking HCV replication [10]. This drug is remarkably free of side effects because human cells have microRNAs other than miR-122 that carry out similar cellular roles (but those other microRNAs do not aid HCV); hence, this RNA antisense drug effectively targets HCV without affecting the host. Furthermore, the inherent sequence-specificity of oligonucleotide drugs that target specific transcripts means that, compared to small-molecule drugs that are capable of potential off-target binding with a diverse range of molecules in cells, oligonucleotide drugs are expected to have fewer side effects and less toxicity.

The first antisense oligonucleotide used as a licensed antiviral drug was Fomivirsen from ISIS (now Ionis) Pharmaceuticals (ISIS-2922, Vitravene), which was approved for use in immunocompromised patients with ocular infections due to human cytomegalovirus (HCMV) [11,12,13] (for a review, see [14]). Fomivirsen prevents the translation of viral protein by direct mRNA binding with key portions of the virus’s genome. The precedents provided by Miravirsen and Fomivirsen clearly show that targeting virus–host RNA–RNA interactions is a valid and relatively untapped approach to antiviral development that provides substantial justification for identifying and validating host RNAs that are essential for viral processes. These precedents suggest that other RNA-based drugs can be developed to target host RNAs that are essential for viral replication, which is the idea that motivated our search for potential host miRNA cofactors of SARS-CoV-2, namely, miRNAs that can be targeted by future sequence-based therapeutics of this type.

Interactions of viruses with host genomic machinery involve complex transcriptional events. In infected hepatocytes, the HCV RNA genome directly interacts with the host miR-122 [9,15]. This pairing is hypothesized to desequester the host miR-122 binding sites transcriptome-wide, in part due to a “sponge” effect [9]. This primes infected cells for oncogenic potential [9], with hepatocellular cancer being a significant HCV comorbidity. We posit that, to facilitate RNA-based drug development for COVID-19, it is essential to identify the potential binding sites (here referred to as “targets”) of host miRNAs on the SARS-CoV-2 virus genome RNA (as well as, potentially, on the SARS-CoV-2-encoded viral messenger RNA transcripts). In this study, we developed a computational meta-analysis tool and resource called miRCOVID-19, which highlights the computationally identified targets of human miRNAs on the SARS-CoV-2 genome. To the best of our knowledge, this is the first report of such a portal. While COVID-19 has only been known for a short time, to date, it has proven refractory to repurposing conventional drugs, including existing antivirals, such as Remdesivir [16,17,18]. We propose miRCOVID-19 as a tool to support and facilitate unorthodox, expedited, sequence-driven discovery and the generation of RNA-based drugs for controlling SARS-CoV-2 and treating COVID-19.

## 2. Materials and Methods

### 2.1. Human MicroRNA Repository

A total of 2638 mature human microRNA sequences were obtained from miRBase v.22 [19]. Among these, 879 were identified as high-confidence mature miRNAs, based on the criteria in miRBase [20].

### 2.2. HCV Genome

For our analysis, we utilized six strains (GenBank accession numbers: NC_004102.1, NC_009823.1, JN588558.1, NC_009825.1, NC_009826.1, NC_009827.1) of HCV representing all six reported genotypes (1–6) of HCV.

### 2.3. SARS-CoV-2 Genome

More than 200 SARS-CoV-2 genome sequences have been deposited in public repositories to date. For our analysis, we utilized six SARS-CoV-2 genomes, which were chosen to represent different locales. The reference genome sequence of SARS-CoV-2 novel coronavirus was obtained from NCBI (GenBank accession no. NC_045512.2). This sequence was isolated in Wuhan, China [21], which is thought to be the point of origin of the pandemic. The other sequences we chose were from Nepal (accession no. MT072688.1), USA (accession no. MT344963.1), Italy (accession no. MT077125.1), South Africa (accession no. MT324062.1), and Brazil (accession no. MT350282.1).

### 2.4. Computational Identification of the Binding Sites of Human miRNAs along the SARS-CoV-2 RNA Genome

To identify the putative binding sites (“targets”) of all human miRNAs on all the analyzed accessions of the SARS-CoV-2 RNA genome (“vRNA”), we used two tools: mirTarP [22] and miRanda [23]. Implementing the mirTarP workflow as published, we followed a two-stage process to identify all putative target sites of human miRNAs along the SARS-CoV-2 genome. In the first stage, we used BLASTN [24] to globally search for nucleotide sequence similarities between mature miRNA sequences and the SARS-CoV-2 genomes. We defined seven consecutive base matches as a minimum seed to identify a BLAST match (BLASTn threshold e-value = 1 × 10^−8^). In the second stage, we used the RNAhybrid [25] tool to find the minimum free energy of formation (MFE) for each BLASTN-predicted miRNA-vRNA hybridization duplex. In parallel with this approach, we also used the miRanda tool to predict the putative target sites of human miRNAs along the SARS-CoV-2 RNA genome. We subsequently focused only on miRNA matches to the SARS-CoV-2 genome that were predicted by both the mirTarP workflow and miRanda, because the computationally independent validation of those matches by the two different approaches increased our confidence in them.

### 2.5. Benchmarking of the Predicted miRNA Targets in the SARS-CoV-2 RNA Genome

Computational predictions inherently carry appreciable false positive and false negative error rates. To reduce the impact of this problem, we limited our analysis to only those predictions that can be considered as potential true hits because they exceed the absolute value of the negative (favorable) MFE of an experimentally validated interaction between a human miR and a viral RNA genome, i.e., the miR-122:HCV RNA interaction. Greater negative MFEs (i.e., negative MFEs with a greater absolute value) signify that the predicted interaction is even more energetically favorable than the documented miR-122:HCV RNA pairing, and is hence more likely to occur and to be experimentally validated. As a benchmark value for MFE, we considered the predicted MFEs of miR-122 against HCV because miR-122 is already validated to bind to the HCV RNA genome [9,15]. Any predicted targets of miRNA on SARS-CoV-2 having an MFE value lower (i.e., stronger) than the MFE of miR-122 on HCV, based on both mirTarP and miRanda, were hence considered as potential true positive hits and were subjected to further analysis, including expression profiling to assess their tissue expression profile relevance to COVID-19 pathogenesis (next section). To reduce and prioritize the list of predicted interactions, we considered only miRNAs that were defined as high-confidence by miRBase [20].

### 2.6. Expression Profiling of the Predicted SARS-CoV-2-RNA-Genome-Binding Human miRNAs

To assess the specificity of miRNA in different cell types, we interrogated the expression profile of each miRNA (both the mature miRNA and the precursor miRNA) in the FANTOM5 Consortium RNA-seq data repository (http://fantom.gsc.riken.jp) (accessed on 25 September 2020) and manually noted all organs, tissues, and cell types (where the miRNA was expressed) that are known to be susceptible to SARS-CoV-2 infection and/or pertinent to COVID-19 pathogenesis, including the entire upper and lower respiratory system, ocular and intestinal epithelial cells, endothelial cells, and immune cells (T- and B-lymphocytes, monocytes, macrophages).

### 2.7. Location of the Predicted SARS-CoV-2-RNA-Genome-Binding Human miRNAs

For all the genes from SARS-CoV-2 and HCV, we considered the gene body and the regulatory region of each gene to check their overlap with the predicted binding sites of miRNAs. For the regulatory region, we considered a [−500, +500] region around the transcription start site (TSS) of each gene. We used BEDTools [26] to check the overlap between predicted target locations and the virus genome.

### 2.8. Gene Ontology (GO) and Pathway Analysis of the Proposed miRNAs

GO analysis and pathway analyses (based on the Kyoto Encyclopedia of Genes and Genomes (KEGG)) were performed on the selected 14 human microRNAs using miRNet [27] and DAVID [28] tools. Briefly, we downloaded the target of the selected miRNAs from miRNet. Then, only statistically significant (*p*-value < 0.05 after a false discovery rate (FDR) correction) GO terms and pathways were selected by considering DAVID.

### 2.9. Conserved Structured and Conserved Unstructured Regions of SARS-CoV-2

We considered the proposed conserved structured and conserved unstructured regions of SARS-CoV-2 that were identified by Rangan *et al.* [29]. We used BEDTools to check the overlap between the predicted target locations and the conserved structured and conserved unstructured regions of the SARS-CoV-2 genome.

## 3. Results

In this study, we first focused on the genomes of all six known human hepatitis C virus (HCV) genotypes. We performed an unbiased, genomewide identification of all binding sites of host (human) microRNAs (miRNA)s, extracted from the definitive miRBase catalog, along the HCV genome. We searched for all possible binding sites of all high confidence miRBase miRNAs anywhere in the viral genome. The miRBase miRNAs that are predicted by our computational pipeline to bind the HCV RNA genome with high affinity included, but were not limited to, the known HCV RNA-binding miRNA miR-122. The mean (±standard deviation (SD)) MFE values of high-confidence human miR-122 across all six HCV genotypes were −26.08 (±3.47) kcal/mol and −23.73 (±3.38) kcal/mol for mirTarP and miRanda, respectively (Table S1). Remarkably, miR-122 target sites did not exhibit the strongest MFEs along the HCV RNA genome, while several miRNAs that were not previously characterized in HCV pathogenesis exhibited more favorable HCV RNA genome binding MFEs than miR-122 did.

We then performed the same analysis for multiple SARS-CoV-2 strains, finding that, similarly to HCV, there were multiple miRNAs predicted by our computational pipeline to bind the SARS-CoV-2 RNA genome with high affinity, and that they included, but were not limited to, miR-122. For the SARS-CoV-2 RNA genome, we found that the average values of the MFE for miR-122 target sites, across the six virus isolates analyzed, were -30.9 (±0.0) kcal/mol and −23.77 (±0.00) kcal/mol, for mirTarP and miRanda respectively (Table S2). Accordingly, the mirTarP prediction was that miR-122 binds the SARS-CoV-2 genomic RNA even more strongly than it binds the HCV genomic RNA, with the latter being an experimentally validated and successfully drugged interaction. The miRanda prediction was that miR-122 binds the SARS-CoV-2 and HCV genomes with comparable affinity. This result suggests that anti-miR-122 oligonucleotide-based HCV drugs, such as Miravirsen and its analogs that are being developed by other companies, may have the potential for successful repurposing as anti-COVID-19 drugs, provided that the SARS-CoV-2 genome actually binds to miR-122 and that the interaction is biologically meaningful. In this respect, it is notable that miR-122—in addition to the liver, where it is an HCV RNA binding partner—is expressed in both alveolar epithelial and intestinal epithelial cells, which act as material for SARS-CoV-2 replication (Table S3). Moreover, miR-122 is also expressed (tags per million (TPM) > 1) in renal glomerular endothelial cells, thoracic endothelial cells, CD14+-monocyte-derived endothelial progenitor cells, aortic endothelial cells, and lymphatic endothelial cells (Table S3). These findings are essential for envisioning RNA-based COVID-19 therapeutic strategies, because miR-122 has been already validated as a therapeutic target in HCV treatments, where its antago-miR (Miravirsen) depletes it and thereby precludes it from functioning as positive HCV cofactor that binds and stabilizes the HCV RNA genome, and because miR-122–in addition to its liver expression essential for HCV pathogenesis–is expressed in epithelial cell types that are susceptible to SARS-CoV-2 infection, and in epithelial and endothelial cell types that are directly pertinent to SARS-CoV-2 replication, pathogenesis, symptomatic COVID-19 consequences, and transmission. Therefore, our results indicate that experimental verification and functional interrogation of miR-122 interactions with the SARS-CoV-2 genome is necessary and can, upon successful completion, lead to repurposing Miravirsen as a COVID-19 treatment if it can be delivered to those cell types. Five other miRNAs predicted to bind the SARS-CoV-2 genome. Five other miRNAs (hsa-miR-1307-3p, hsa-miR-766-3p, hsa-miR-1910-5p, hsa-mir-296-3p, hsa-miR-1304-5p) were also expressed (>1 TPM) in almost all the endothelial cells collected in FANTOM. These other miRNAs that we here identified for the first time as SARS-CoV-2 genome binders in relevant cell types and tissues are also strategically important for designing better therapeutics, because–provided that these miRNAs’ interactions with the SARS-CoV-2 genomic RNA can be experimentally verified and functionally validated–their interactions with the viral genome can be targeted by sequence-based RNA drugs, such as antago-miRs, to interfere with viral reproduction and pathogenesis. For SARS-CoV-2, we considered only miRNAs targets that had a minimum MFE that was more favorable than the MFE for the predicted miR-122:SARS-CoV-2 RNA interaction. Additionally, if targets are selected by both tools in at least two strains of SARS-CoV-2, we only considered them as potential targets of miRNA on SARS-CoV-2 (Table S2). In Table 1, we present the list of miRNAs meeting all these criteria.

Interestingly, the predicted targets (binding sites) of the host miRNAs on viral genomes (both HCV and SARS-CoV-2) were not limited to gene regions and were characterized by a high diversity of genomic locations (Table S4). These included the gene bodies of viral genes, as well as the regulatory regions of the genes and the viral untranslated regions (UTRs). In both HCV and SARS-CoV-2, genes account for most of the genome, while intergenic regions are scant. Therefore, the promoter regions of genes occupy only a limited space. As a result of the compact, dense, and gene-rich structure of these two viral genomes, the number of miRNA targets in the gene bodies was higher compared to that in the promoter regions of the viral genes. Based on the overlap of the predicted targets and the different regions of SARS-CoV-2, we noticed that the predicted targets of human miRNAs occurred in diverse locations in the genome, including in the genes encoding the structural proteins, as well as in the genes for nonstructural proteins (Nsps). Out of the four structural protein genes (envelope (E), membrane (M), nucleocapsid (N), and spike (S) proteins) of SARS-CoV-2, we found the target of miRNAs on three of them: M, N, and S. We did not find any predicted host microRNA target hits on the E protein gene. Out of the 16 Nsps (Nsp1–Nsp16) from SARS-CoV-2, both tools predicted the targets of high-confidence miRNAs as being on the Nsp2 and Nsp3 genes. Additionally, miRanda also predicted targets for hsa-miR-187-5p, hsa-miR-885-3p, hsa-miR-1304-5p, hsa-miR-1912-5p, and hsa-miR-4761-5p on Nsp4, Nsp6, Nsp7, Nsp8, Nsp9, and Nsp10. Both tools predicted that hsa-miR-1307-3p should bind the UTR of SARS-CoV-2.

We also checked the predicted miRNA target regions against the conserved structured and conserved unstructured regions of SARS-CoV-2. For three miRNAs, namely, hsa-miR-122-5p, hsa-miR-1304-5p, and hsa-miR-885-3p, the targets predicted by both tools (miRanda and mirTarP) were in the conserved structured region of SARS-CoV-2 (Table S5). For seven other miRNAs (hsa-miR-598-5p, hsa-miR-6834-5p, hsa-miR-514b-5p, hsa-miR-1910-5p, hsa-miR-766-3p, hsa-miR-1912-5p, hsa-miR-187-5p), the targets predicted by at least one of the tools were in the conserved structured region. The conserved structured region could be considered as a candidate for small molecule drug therapeutics. For two miRNAs, namely, hsa-miR-296-3p and hsa-miR-1304-5p, the target regions predicted by the miRanda tool were in the conserved unstructured region of SARS-CoV-2. These two targets, if they can be experimentally validated, should be considered for the binding of antisense oligonucleotides to modulate the interaction of the SARS-CoV-2 genome with these miRNAs. For two miRNAs, namely, hsa-miR-1304-5p and hsa-miR-1307-3p, the predicted target region was in the 5′ and 3′ UTRs, respectively.

The selected miRNAs are involved in different KEGG pathways pertaining to cancer, including glioma, leukemia, melanoma, bladder cancer, and proteoglycans, as well as controlling the cell cycle (Table 2). This is potentially interesting and provocative because HCV infection of the liver is well known to result in a predisposition to liver cancer (hepatocellular carcinoma). COVID-19 is a newly emerging disease, hence insufficient time has elapsed to date to map its long-term consequences; since numerous other viruses increase susceptibility to specific cancers, and in view of the SARS-CoV-2 genome’s predicted interactor miRNAs’ gene ontology hits, cancer incidence in COVID-19 survivors should be considered in longitudinal follow-ups.

From the molecular function module of the GO analysis, we found that 14 high-confidence miRNAs were involved in different binding and adhesion activities, including DNA binding, RNA binding, nucleoside binding, and protein binding in cell adhesion. From the cellular component module of the GO analysis, we inferred that our 14 high-confidence miRNAs were involved in focal adhesion, cell–cell junctions, and cell–cell adherence junction. This further explains the role of these miRNAs in the signaling process and cancer metastasis [30]. Table S6 highlights all the GO terms and pathways that were significantly associated with the 14 high-confidence miRNAs.

In summary, we have identified 14 miRNAs that had more favorable MFEs for their predicted interactions with the SARS-CoV-2 genome, by both mirTarP and miRanda, than the MFE (derived by the same methods) of the experimentally validated, functional, and druggable miR-122:HCV RNA genome interaction. The interactions of these 14 miRNAs with the SARS-CoV-2 RNA genome should be considered for experimental validation. If the interactions can be validated, if they occur in cell types pertinent to COVID-19 pathogenesis, and if they are proven to be functionally relevant to SARS-CoV-2 infection of these cells, then development (or, in the case of miR-122, repurposing) of anti-miR drugs to inhibit these interactions is recommended. Crucially, all 14 miRNAs are expressed in cells and tissues that are either known to be susceptible to infection by SARS-CoV-2 or are relevant to COVID-19 pathogenesis.

## 4. Discussion

The first surprising outcome of our analysis was that miR-122 exhibited more favorable MFEs for SARS-CoV-2 genomic RNA binding, according to both mirTarP and miRanda, than for its experimentally validated, successfully drugged HCV genomic RNA binding. Although miR-122 expression was initially reported in hepatocytes, the FANTOM5 Consortium expression datasets indicate that miR-122 is also expressed in intestinal epithelial cells (Table S3), which is an important finding in view of the recent observations of robust SARS-CoV-2 fecal shedding in infected individuals, particularly pediatric patients, and speculations of oral–fecal transmission compounding the known respiratory transmission of this novel pathogen, which is a virus whose easy qRTPCR-based detection in sewage is now serving as a key method of quantifying the spread of the pandemic, as well as assessing its early history [31,32]. These observations, and the fact that miR-122 is expressed at higher levels in many COVID-19-relevant cell types than it is in hepatocytes where its HCV RNA binding was elucidated, imply that miR-122 should be experimentally tested as a plausible cellular co-factor of SARS-CoV-2 infection, and that, if such a link is validated, the existing Roche/Santaris oligonucleotide anti-HCV miR-122-antisense drug Miravirsen (and its chemical analogs, developed subsequently by Regulus Therapeutics [33]) should be tested for possible repurposing as an anti-SARS-CoV-2 therapeutic agent.

Although miRs canonically serve as post-transcriptional repressors of cellular mRNAs, they may have unconventional roles as activators, for example, in the case of the miR-122:HCV relationship. One possibility is that miR-122 interacts with the SARS-CoV-2 RNA genome in the same way that it interacts with the HCV genome, and the host–virus RNA–RNA binding facilitates viral replication and function. The second possibility is that miR-122 is a suppressor of SARS-CoV-2. Our bioinformatics analysis suggests that the potential for miR-122:SARS-CoV-2 genome RNA:RNA binding is comparable to, or higher than, that for miR-122:HCV genome RNA:RNA binding. Since the latter has been validated in the laboratory and drug-targeted, our analysis should guide and motivate future laboratory-based validation of the miR-122:SARS-CoV-2 genome RNA:RNA binding. If this RNA–RNA interaction can be validated in infected cells, its functional potential should then be assessed and, if present, it should be drug-targeted, i.e., antagonized if the miR turns out to be a positive cofactor of SARS-CoV-2 or enhanced if the miR turns out to be an antagonist of the virus. In addition to this miR-centric model of the host–viral RNA–RNA interaction, it is possible that the SARS-CoV-2 genome, especially in high copy numbers in infected cells, may sequester cellular miRNAs during pathogenesis; if that is the case and the miRNAs are protective or viral antagonists, they should be enhanced or rescued in therapies. All these scenarios may be compounded by RNA–protein interactions, such as those that obstruct regions of the viral RNA, making them inaccessible for host miRNA binding; the competition between miRNAs and RNA-binding proteins for the same mRNA [34] or viral RNA targets; the sequestering of cellular miRNAs, making them incapable of hybridizing to their cognate sites along the viral genome. These scenarios make the experimental validation of our predicted, therapeutically targetable host–virus RNA–RNA interactions even more of a biological imperative.

Our second contribution to the field was the identification of additional miRs that are likely to bind the SARS-CoV-2 RNA genome. All the miRs identified were expressed in respiratory epithelial cells, namely, small-airway, bronchial, alveolar, tracheal, olfactory, and/or nasal epithelial cells, depending on the individual miR (Table S3). Some of the miRs (hsa-miR-1307-3p, hsa-miR-1912-5p, hsa-miR-766-3p, hsa-miR-1910-5p, hsa-miR-1304-5p) were also expressed in lung fibroblasts (Table S3). This prevalent respiratory epithelial expression of miRs, which were predicted by two independent tools to bind the SARS-CoV-2 genome with a stronger affinity than the documented benchmark miR-122:HCV interaction, indicates—given SARS-CoV-2’s propensity to infect and replicate in these cell types and tissues—the potential for molecular interactions between the viral genome and these microRNAs in infected cells, and the need to interrogate any such interactions for their contribution to viral replication and COVID-19 pathogenesis. We recognize that, even if the interactions are validated and are functional in COVID-19 pathogenesis, the delivery of the anti-miRs to the infected cells might represent a challenge. The anti-miR-122 HCV treatment works well in part because of the natural propensity of modified-backbone oligonucleotides that are intravenously injected into the bloodstream to efficiently target the liver [35]; in contrast, anti-miRs targeting the respiratory system tissues infected by SARS-CoV-2 may be more challenging, although it likely is feasible, as extensive proof-of-principle work on small-RNAs targeting these tissues, including via inhalation, has already been performed [36].

Some of the 14 miRs found were also expressed in immune system or immunity-associated cell types, such as macrophages (hsa-miR-1307-3p, hsa-miR-1912-5p, hsa-miR-766-3p, hsa-miR-1304-5p), mast cells (hsa-miR-1307-3p, hsa-miR-1912-5p, hsa-miR-766-3p), and T-cells (has-miR-122-5p, hsa-miR-1304-5p). In view of the recent finding that SARS-CoV-2 can infect T-cells, although it is unable to replicate in them [37], and in view of the rising importance of T-cell-mediated immunity in contrast to conventional antibody-mediated immunity in the immune response to SARS-CoV-2 infection [38], these expression profiles indicate additional possible venues for the interactions of these miRs with the viral genome or virally encoded transcripts.

A further important observation was that all of the identified miR–viral genome interactions were predicted to occur with equal or nearly equal minimum free energies of formation (all of which exceed our thresholds and were validated by both of the tools that we applied) between all the miRs and all six SARS-CoV-2 variants (strains) that we sampled. While over 200 SARS-CoV-2 variants are now in the public domain and it is possible that some of them might have substitutions that would abolish these interactions, this result is expected because the evolutionary origin of SARS-CoV-2 is so recent [39] that there has not yet been sufficient time for a meaningful divergence of the different isolates. At the same time, our result is promising in terms of treatment development because the identified miR–viral RNA genome interactions are a common property of all tested extant variants of SARS-CoV-2 and are not strain-specific; therefore, any successful antago-miR drug would be equally efficient against all SARS-CoV-2 strains, not just some of them, just as Miravirsen is efficient against all HCV genotypes because the sequence of its binding site along the HCV genome is conserved in all of them.

## 5. Conclusions

Our results indicate the need to test whether any of the 14 identified miRs interact with SARS-CoV-2 genomic RNA or transcripts in infected cells, and to determine whether these interactions are relevant to pathogenesis and viral replication using established methodologies involving in vitro infected cell culture systems, such as viral plaque formation assays. Any pathogenesis-relevant interactions should subsequently be tested as targets for miRNA-antisense (antago-miR) Miravirsen-type drug candidates, and the effects of such candidates on viral replication can subsequently be explored for developing the candidates into therapeutic leads. Furthermore, if this COVID-19 RNA drug development pipeline emerges as a productive path for treating this newly emerged disease, our insights can be readily adapted for developing antago-miR therapies against the numerous other RNA-genome viruses of great public health significance that cause currently untreatable diseases.

## Figures and Tables

**Table 1:** Summary of the minimum free energy of formation (MFE; mean ± SD) for the 14 “high-confidence” microRNAs (miRNAs) from miRBase with potential target sites on severe acute respiratory syndrome coronavirus 2 (SARS-CoV-2)

miRNA	Accession No.	mirTarP	miRanda
hsa-miR-122-5p	MIMAT0000421	−30.9 ± 0	−23.77 ± 0
hsa-miR-766-3p	MIMAT0003888	−27.98 ± 2.72	−25.22 ± 0
hsa-miR-1910-5p	MIMAT0007884	−27.96 ± 3.14	−26.07 ± 0
hsa-miR-4761-5p	MIMAT0019908	−29.3 ± 3.176	−29.43 ± 0
hsa-miR-296-3p	MIMAT0004679	−29.83 ± 1.65	−29.19 ± 1.46
hsa-miR-598-5p	MIMAT0026620	−31 ± 0.464	−24.7 ± 0
hsa-miR-885-3p	MIMAT0004948	−30.066 ± 1.290	−27.78 ± 0
hsa-miR-6834-5p	MIMAT0027568	−31.083 ± 3.283	−30.56 ± 0
hsa-miR-187-5p	MIMAT0004561	−31.3 ± 0	−29.62 ± 0
hsa-miR-149-3p	MIMAT0004609	−31.8 ± 0	−23.98 ± 0
hsa-miR-1304-5p	MIMAT0005892	−32 ± 0	−28.1 ± 30
hsa-miR-1307-3p	MIMAT0005951	−37.6 + 0	−33.56 ± 0
hsa-miR-1912-5p	MIMAT0037333	−32 ± 0	−27.25 ± 0
hsa-miR-514b-5p	MIMAT0015087	−31 ± 0	−26.82 ± 0

**Table 2:** Summary of Kyoto Encyclopedia of Genes and Genomes (KEGG) pathways for the 14 “high-confidence” miRNAs from miRBase with potential target sites on SARS-CoV-2.

KEGG Term	Description	*p*-Value	FDR
hsa05214	Glioma	7.6400 × 10^−5^	0.0164
hsa04115	p53 signaling pathway	1.2500 × 10^−4^	0.0164
hsa04066	HIF-1 signaling pathway	7.4300 × 10^−4^	0.0491
hsa04550	Signaling pathways regulating pluripotency of stem cells	8.1000 × 10^−4^	0.0491
hsa05220	Chronic myeloid leukemia	1.0337 × 10^−3^	0.0491
hsa05205	Proteoglycans in cancer	1.1209 × 10^−3^	0.0491
hsa05230	Central carbon metabolism in cancer	1.5538 × 10^−3^	0.0497
hsa04152	AMPK signaling pathway	1.6337 × 10^−3^	0.0497
hsa04110	Cell cycle	1.8644 × 10^−3^	0.0497
hsa05219	Bladder cancer	2.1258 × 10^−3^	0.0497
hsa05218	Melanoma	2.2046 × 10^−3^	0.0497
hsa04012	ErbB signaling pathway	2.2693 × 10^−3^	0.0497
hsa04919	Thyroid hormone signaling pathway	2.4828 × 10^−3^	0.0502

FDR: false discovery rate.

## Data Availability

The data presented in this study are available on request from the corresponding author.

## Supplementary Materials

The following are available online at https://www.mdpi.com/2311-553X/7/1/18/s1.

Table S1: Summary results of miR-122 target sites on the hepatitis C virus (HCV) RNA genome using mirTarP and miRanda tools.

Table S2: Summary results for high-confidence miRNAs (from miRBase) and minimum free energy of formation (MFE) on multiple strains of SARS-CoV-2 based on mirTarP and miRanda tools.

Table S3: Expression profile of the selected high-confidence miRNAs (from miRBase) in FANTOM5 cell types.

Table S4: List of 14 high-confidence human miRNAs, their predicted target location, and their MFEs as predicted using mirTarP and miRanda tools.

Table S5: List of 14 high-confidence all-human miRNAs and their predicted target locations in the conserved structured and conserved unstructured region of severe acute respiratory syndrome coronavirus 2 (SARS-CoV-2).

Table S6: Functional analysis (Gene Ontology (GO) term and pathways) of the proposed 14 human miRNAs using miRNet and DAVID.
